# Calcium alginate entrapped *Eupatorium adenophorum* Sprengel stems powder for chromium(VI) biosorption in aqueous mediums

**DOI:** 10.1371/journal.pone.0213477

**Published:** 2019-08-16

**Authors:** Mahendra Aryal

**Affiliations:** Department of Chemistry, Tri-Chandra Multiple Campus, Tribhuvan University, Kathmandu, Nepal; King Saud University, SAUDI ARABIA

## Abstract

A novel biosorbent, *Eupatorium adenophorum* Sprengel-alginate beads was used for chromium(VI) biosorption from aqueous solutions. Biosorption process was optimized at pH 2.0, biomass concentration 1.0 g/L, contact time 60 min, and temperature 30 ^o^C respectively. Maximum uptake capacity of Cr(VI) was calculated at 28.011 mg/g. It was found that the overall biosorption process was best described by pseudo second-order kinetics with high correlation coefficient values. Intraparticle diffusion model suggested that Cr(VI) biosorption may proceed within multiple steps. Data obtained from the batch studies confirmed well to the Langmuir, Temkin, and Hill-der Boer isotherm models. Scatchard plot analysis further supported the mono-layer biosorption of Cr(VI) ions on *Eupatorium adenophorum* Sprengel-alginate beads as described by Langmuir isotherm model. Numerical values of *E* obtained from Dubinin-Radushkevich isotherm model identified the physisorption as predominant mechanism for Cr(VI) biosorption. The negative values of Δ*G*^*o*^ confirmed the spontaneous and feasibility nature, whereas positive value of Δ*H*^*o*^ showed the endothermic nature of biosorption process. Positive value of Δ*S*^*o*^ indicated an increase in the randomness at the solid/solution interface during the biosorption process. The endothermic nature of Cr(VI) biosorption was also described by Temkin isotherm model. The results indicated that Cr(VI) biosorption was not significantly affected by the presence of co-ions at lower concentrations. Desorption of Cr(VI) ions from metal-loaded *Eupatorium adenophorum*-alginate beads was observed at 92.091% with 0.5 M HNO_3_ solution in solid to liquid ratio of 1.0 g/L.

## Introduction

Chromium is a potentially toxic metal originating from anthropogenic activities such as the mining of chromium ores, iron-steel, electroplating, tanning, printing, dyeing, papermaking, and textile industries, municipal waste landfill, and sewage irrigation, pesticide, herbicides, antibiotics, and fertilizer application [1,2,3,4]. Such numerous industries have resulted in a generation of large quantities of liquid effluent loaded with high concentration of chromium. It is generally found as trivalent and hexavalent states in aqueous environments [1,5]. More specifically, Cr(III) is relatively non-toxic and required in micro quantities in human food [6]. Under certain conditions, Cr(III) can be oxidized to more carcinogen and mutagen Cr(VI) in the environment [4,5]. On contrary, Cr(VI) can persist in the long-term in soil and wastewater because of its non-biodegradability nature [4]. It is known that Cr(VI) is 100 times more toxic than Cr(III) [1]. Cr(VI) is the most stable species in drinking water, and it can have lethal effects on human physiological, neurological and biological systems [1,2,3]. Therefore, the maximum permissible limit of Cr(VI) ions in drinking water has been proposed at 0.1 mg/L **[7]**.

The quantity of Cr(VI) that exists in industrial effluents released into the environment is often higher than the acceptable level. Hence, attention should be made to reduce its quantities from effluents by suitable treatment technologies before it is discharged into the natural environment [8]. Different treatment technologies such as precipitation, coagulation, ion exchange, adsorption, oxidation, reduction, and reverse osmosis techniques have been applied in order to remove the chromium species from contaminant streams [2]. Each of the methods exhibits shortcomings such as high operational cost, low removal efficiency, difficult operation process, limited tolerance to pH change, secondary pollution, and ineffective for small scale industries [4,9,10]. Due to this reason, there is an immediate action for suitable and cost effective removal technology for heavy metals [4]. Recently, biosorption has become one of the alternative methods that has been widely applied for detoxification of heavy metals [1,2]. It has many advantages over conventional methods such as low cost, easy operating system, high removal efficiency, removal of metal ions at low concentrations, high metal binding ability, low biological sludge formation, eco-friendly, recycling of biosorbents, and high recovery of metal ions from metal-loaded biosorbents [2,6,8].

The bio-materials such as algae, fungi, yeast, bacteria, agricultural and industrial by-products have been studied for removal of heavy metals [1,8,11]. It is reported that biomaterials have a high potential for heavy metal removal [9,12]. Low cost biosorbents are becoming the focus of many researches [4]. The plant biosorbents including *Acacia albida* and *Euclea schimperi* [13], *Cicer arientinum* [14], *Cupressus lusitanica* bark [10], eucalyptus bark [15], *helianthus annuus* stem waste [16], neem sawdust and mango sawdust [17], mango peels [2], *Plataneusorientalis* leaves [18], *Polyporus squamosus* [19], *Senna siamea* [6], *Stipa tenacissima* [3], *Ulmus* leaves [20], and wheat bran [21] etc. have been investigated for removal of Cr(VI) ions from aqueous medium.

Application of native or free biosorbents for removal of heavy metals has some limitations including low density and mechanical strength, a wide particle size distribution, and poor isolation of solid and liquid phases [22,23,24]. The several matrices have been employed in immobilization of the biosorbents for heavy metal removal in order to overcome such limitations [25]. The immobilization of biomaterials is very important step for large scale-up of effluents treatment, and its main advantages include an increase in the density and mechanical strength of the biomass, easy isolation of solid and liquid phases, retention of biomass within the reactor [23,25]. Use of immobilized biomaterials in industrial scale can reduce separation costs by 60% [23]. Entrapment of biosorbents in the alginate has been used in heavy metal removal, due to its ease of preparation, biodegradability, hydrophilicity and softness in nature with high mechanical strength. Alginate is a component of the outer cell wall of brown algae and certain bacteria. Due to the availability of carboxyl groups, immobilization with alginate can enhance the efficiency of biosorption of heavy metals [24].

To the best of our knowledge, *Eupatorium adenophorum* Spreng. stems biomass entrapped in calcium alginate has not yet been used for Cr(VI) biosorption, thus, it was finally selected to study in Cr(VI) biosorption. Kinetics, isotherm and desorption studies as well as effect of interfering ions on Cr(VI) biosorption were studied. The main aim of this research work was to improve the sequestration potential of immobilized *Eupatorium adenophorum* biomass for removing Cr(VI) ions from aqueous solutions.

## Materials and methods

### Ethics statement

*Eupatorium adenophorum* Spreng. (Syn. *Ageratina adenophorum*) is not endangered species and no specific permission is required for harvesting from the non-protected area in the purpose of scientific research activities. No material was taken from protected area or national park. Author used stems of this plant that came from his own garden.

### Collection of biosorbent

**Fig 1:**
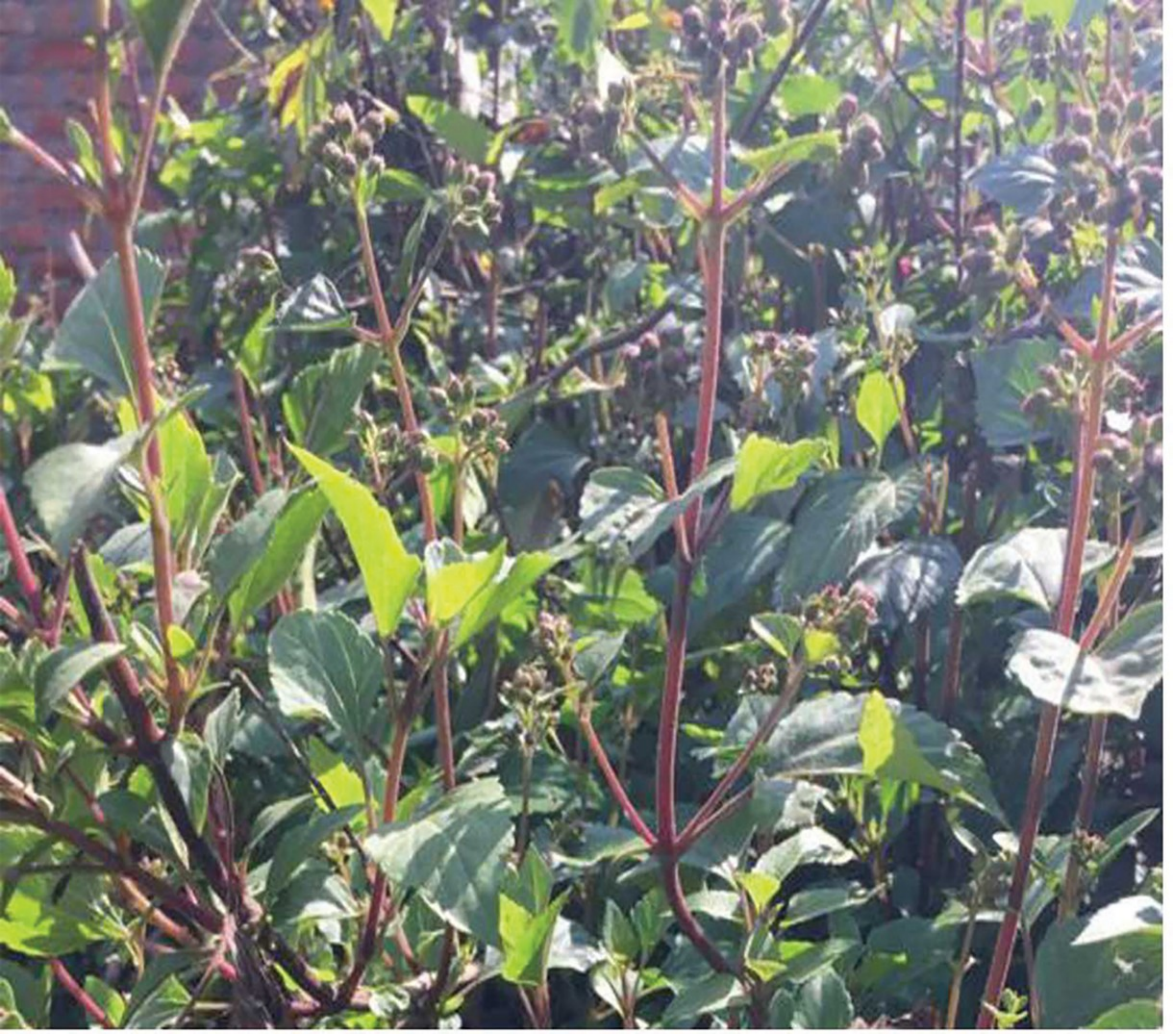
*Eupatorium adenophorum* Sprengel (Banmara) plant.

This plant is a widely growing and spreading perennial herbaceous shrub that may grow to 1 or 2 m high, and can cause to damage the grazing land and natural forest (Fig 1). It is locally known as banmara (killer of the forests) [26]. It was collected from Besisahar Municipality-9, Nepal. The study site is located at Pakhathok (Latitude 28^o^ 13' 42'' N and Longitude 84^o^ 22' 22'' E) with an altitudinal range of 1003 m. Annual average temperature and precipitation/rainfall are 13.6–25.3°C and 20–762 mm respectively. *Eupatorium adenophorum* Spreng. was indentified according to the criteria as described by Wolff, [27].

### Preparation of biosorbent

*Eupatorium adenophorum* Spreng. stems were first cut into small pieces and dried in sunlight until all the moisture was evaporated, and then converted into powdered form. After being powdered, it was treated with 1.0 M HCl in order to remove the impurities as well as to expose more binding sites. It was then washed with distilled water as long as the pH of the washing solution becomes in neutral range. After washing, moisture content of biomass was determined by drying a pre-weighted amount in hot air oven (Accumax India) at 100 ^o^C for 24 h.

### Immobilization of biosorbent

Calcium alginate was used as an immobilizing agent for *Eupatorium adenophorum* Spreng.stems powder. In this method, two percentage of sodium alginate was first dissolved in distilled water, and it was mixed with calculated amount of dried *Eupatorium adenophorum* stems powder. *Eupatorium adenophorum*-alginate mixture was then dropped by a syringe into the 5% CaCl_2_ solution placed on magnetic stirrer held around 5–8°C. The beads were hardened in this solution for 24 h in refrigerator at 4 ^o^C [11]. The beads were washed with distilled water for several times to remove excess of calcium ions and un-trapped particles of *Eupatorium adenophorum* Spreng. biomass. Thus, obtained beads were then air dried. It was found that 1.0 g of alginated beads contains 0.052 g of dry biomass. The immobilized biomass was used for further biosorption experiments.

### Preparation of stock metal solution

Stock metal solution was prepared by dissolving the calculated amounts of K_2_Cr_2_O_7_ (Merck, Germany) in distilled water to obtain the standard solutions of 1000 mg/L of Cr(VI) ions. The desired metal solutions were prepared by dilution of the stock standard solution.

### Biosorption experiments

Biosorption experiments were carried out in 50 ml conical flasks. Initial Cr(VI) concentration of 10 mg/L was used in order to determine the optimum pH, contact time, biomass concentration and temperature. The Cr(VI) biosorption on *Eupatorium adenophorum-*alginate beads was performed with varying initial pH values from 1.0 to 7.0, biomass concentration from 1.0 to 7.0, contact time from 0 to 130 min, and temperature from 20 to 40°C respectively. Equilibrium isotherm experiments were carried out with initial Cr(VI) concentration between 10 and 300 mg/L at pH 2.0, biomass concentration 1.0 g/L and contact time 60 min respectively. All experiments were performed in triplicate and the mean values were used in the data analysis. The Cr(VI) concentration was determined at 540 nm followed by complex formation with 1,5-diphenylcarbazide (Merck, Germany) using a spectrophotometer (Shimadzu, Japan) [28].

### Effect of interfering ions on Cr(VI) biosorption

Standard solutions of SO_4_^-2^, Cl^-^, CO_3_^-2^, Mg^+2^, Ca^+2^, Fe^+3^, Zn^+2^, Cd^+2^, Cu^+2^ and Ni^+2^ were prepared from Na_2_SO_4_, NaCl, Na_2_CO_3_, MgSO_4_.7H_2_O, CaCl_2_.2H_2_O, FeCl_3_.7H_2_O, ZnSO_4_.7H2O, CdSO_4_.H_2_O, CuSO_4_.5H_2_O and NiSO_4_.6H_2_O respectively. The effect of interfering ions ranging from 5 to 50 mg/L on Cr(VI) biosorption at 10 mg/L of Cr(VI) ions was conducted.

### Desorption studies

Biosorption experiments were first conducted with initial Cr(VI) concentrations of 250 mg/L at optimum conditions of pH, biomass concentration, contact time and temperature respectively. After Cr(VI) biosorption, *Eupatorium adenophorum*-alginate beads were collected carefully and washed with distilled water. The Cr(VI) ions-adsorbed beads were dried in oven at 60 ^o^C for 24 h and then again suspended in 30 ml of 0.5 M HNO_3_ solution at room temperature for 2 h.

### Data analyses

A number of mathematical models have been employed to analyze the experimental data in order to establish the extent of biosorption of heavy metals. The amount of Cr(VI) ions adsorbed by *Eupatorium adenophorum*-alginate beads is given by the following equation;

$$Q_{e}=\frac{V.(C_{o}-C_{e})}{W}$$ (1)

Where *Q*_e_ is the amount of Cr(VI) adsorbed by the biomass (mg/g) at equilibrium, *C*_o_ is the initial concentration of Cr(VI) (mg/L), *C*_e_ is the concentration of Cr(VI) at equilibrium (mg/L), *V* is the initial volume of Cr(VI) solution (L), and *W* is the mass of the biosorbent (g).

### Langmuir-Hinshelwood (L-H) kinetic model

Langmuir-Hinshelwood (L-H) kinetic model has been applied to determine the adsorption kinetics of metal ions on biomass surface [29].

$$\frac{\ln(\frac{C_{o}}{C_{M}})}{\left(C_{o}-C_{M}\right)}+k_{o}=k_{1}.\frac{t}{\left(C_{o}-C_{M}\right)}$$ (2)

Where *k*_*o*_ is similar to a zero-order rate constant that represents the initial sorption rate (L/mol), *k*_*1*_ is first-order rate constant after sorption reaches its maximum (min^-1^) and *C*_*M*_ is metal ion concentration in solution (mol/L). The values of *k*_*1*_ and *k*_o_ can be determined from the slope and intercept of a plot between ln(*C*_o_/*C*_M_)/(*C*_o_-*C*_M_) and *t*/(*Co*-*C*_*M*_) respectively.

### Avrami kinetic model

This kinetic model describes the fractionary kinetic orders. Some parameters of this model indicate the possible changes in the sorption rates as function of the initial metal ion concentration and adsorption time [30].

$$\ln\left\{-\ln(1-\alpha )\right\}=n\ln k_{av}+n\ln t$$ (3)

Where *α* is an adsorption fraction (*Q*_t_/*Q*_e_) at time *t*, *k*_*av*_ is the kinetic constant (min^-1^) and *n* is a fractionary kinetic order. The values of *n* and *k*_*av*_ can be determined from slope and intercept of a plot of ln{−ln(1 - α)} and ln*t* respectively.

### Lagergren pseudo-first order kinetic model

According to this kinetic model, the rate of occupation of binding sites is proportional to the number of unoccupied sites [31].

$$\ln(Q_{e}-Q_{t})=\ln Q_{e}-k_{{}_{1}}.t$$ (4)

Where *k*_1_ is the pseudo first order rate constant (min^-1^). The values of *Q*_*e*_ and *k*_*1*_ can be obtained from the slope and intercept of a plot between ln(*Q*_*e*_*−Q*_*t*_) and *t* at different temperatures.

### Ho pseudo-second order kinetic model

It considers that the rate of occupation of binding sites is proportional to the square of the number of unoccupied sites [32].

$$\frac{t}{Q_{t}}=\frac{1}{k_{2}Q_{e}{}^{2}}+(\frac{1}{Q_{e}}).t$$ (5)

Eq (5) can be further written as:

$$\frac{t}{Q_{t}}=\frac{1}{h}+\frac{t}{Q_{e}}$$ (6)

If pseudo-second order kinetic is applicable, the plot of *t*/*Qt* versus *t* gives a straight line and its slope and intercept give the values of *k*_2_ or *h*, and *Q*_*e*_ respectively.

### Ritchie pseudo second-order kinetic model

This kinetic model describes that one adsorbate is adsorbed on two binding sites and the rate of adsorption depends solely on the fraction of the binding sites of adsorbents [33].

$$\frac{1_{}}{Q_{t}}=\frac{1}{k_{r}Q_{e}t}+\frac{1}{Q_{e}}$$ (7)

Where *k*_*r*_ is rate constant of the Ritchie-second order kinetic model (1/min). The value of *k*_*r*_ can be determined from the slope of a plot between 1/*Q*_*t*_ and 1/*t* respectively.

### Sobkowsk and Czerwinski pseudo-second order kinetic model

This kinetic model purposes that first order reaction can be applied for lower surface concentrations of solid and the second order reaction for higher surface concentrations [34].

$$\frac{\theta }{1‐\theta }=k_{2}t$$ (8)

Where *θ* (*Q*_t_/*Q*_*e*_) is the fraction of surface sites, which are occupied by adsorbed metal ions. The second order rate constant, *k*_*2*_ (min^-1^) can be calculated from a straight line obtained from the plot of *θ*/(1-*θ*) against *t* respectively.

### Blanachard pseudo-second order kinetic model

Blanachard second order kinetic model initially proposed similar to that of Ritchie’s model for the exchange reaction of divalent metallic ions onto NH_4_^+^ ions in fixed zeolite particles [35].

$$\frac{1}{Q_{e}{‐Q}_{t}}-\alpha =k_{2}t$$ (9)

The rate constant, *k*_*2*_ (g/mg.min) can be evaluated from the slope of a plot between 1/(*Q*_*e*_-*Q*_t_) versus *t* respectively.

### Elovich kinetic model

Elovich model can be used to evaluate the kinetics of chemisorption of adsorbate onto heterogeneous adsorbent surface [36].

$$Q_{t}=\frac{1}{\beta }\ln(\alpha \beta )+\frac{1}{\beta }\ln t$$ (10)

Where *α* is the initial adsorption rate (mg/g·min), and *β* is the extent of surface coverage and activation energy (g/min). If the biosorption data correlate with Elovich equation, a plot of ln*t* verses *Q*_*t*_ gives a straight line and the values of *β* and *α* can be determined from its slope and intercept respectively.

### Intraparticle diffusion kinetic model

It explains the diffusion mechanism of the biosorption process [37].

$$Q_{t}=k_{id}.t^{0.5}+C$$ (11)

The value of intraparticle diffusion rate constant (*k*_*id*_) can be obtained from the slope of a linear plot of *Q*_*t*_ versus *t*^*0*.*5*^, whereas *C* is the intercept.

### Langmuir isotherm model

According to the Langmuir isotherm model, there are a finite number of binding sites, which is mono-layer coverage due to homogeneously distributed over the adsorbent surface and there is no interaction between adsorbed metal ions [38].

$$\frac{C_{e}}{Q_{e}}=\frac{C_{e}}{Q_{\max}}+\frac{1}{Q_{\max}.b}$$ (12)

Where *Q*_max_ is the maximum uptake capacity (mg/g) and *b* is the binding affinity (L/mg). The adsorption feasibility can be evaluated by the Langmuir isotherm separation factor, *K*_*L*_.

$$K_{L}=\frac{1}{\left(1+b.c_{e}\right)}$$ (13)

The values of *K*_L_ in the range 0 to1 indicate the favorable uptake of metal ions [39].

### Scatchard plot analysis

The best fit of Scatchard analysis with experimental equilibrium data suggests that the single or distinct types of binding sites, which is an indication of mono-layer coverage [40].

$$\frac{Q_{e}}{C_{e}}=Q_{\max}.b-Q_{e}.b$$ (14)

The values *Q*_*max*_ and *b* can be determined from the slope and intercept of a plot between *Q*_*e*_/*C*_*e*_ and *Q*_*e*_ respectively.

### Freundlich isotherm model

Freundlich isotherm model explains about the multi-layer adsorption of metal ions on heterogeneous adsorbent surface [41].

$$\ln Q_{e}=\ln K_{f}+(\frac{1}{n})\ln C_{e}$$ (15)

Where *K*_*f*_ and *n* are the biosorption capacity and intensity respectively. High values *K*_*f*_ and 1/*n* suggest that there is a strong affinity between metal ions and surface binding sites.

### Gin isotherm model

Gin et al., [42] proposed a simple equilibrium isotherm model, which can be described by following linear equation;

$$\ln\frac{C_{e}}{C_{o}}=\frac{1}{C_{o}}(\beta M)+\ln\alpha$$ (16)

The value of *β* and *α* can be calculated from the slope and intercept of a plot between ln (*C*_*e*_/*C*_*o*_) and 1/*C*_*o*_ respectively.

### Temkin isotherm model

This model describes the behavior of adsorption systems on heterogeneous biomass surfaces. It tells that the heat of sorption of all molecules in a layer decreases linearly due to the adosorbent-sorbate interaction [43].

$$Q_{e}=B_{T}\ln a_{T}+B_{T}\ln C_{e}$$ (17)

Where;

$$B_{T}=\frac{RT}{b_{T}}$$

Where *T* is the absolute temperature (K), *R* is the universal gas constant (kJ/mol), *b*_*T*_ is the heat of adsorption (kJ/mol), and *a*_*T*_ is equilibrium binding constant (L/mol) respectively.

### Dubinin-Radushkeich (D-R) isotherm model

It indicates the heterogeneity of the surface energies [44].

$$\ln Q_{e}=\ln Q_{m}-\beta .\varepsilon^{2}$$ (18)

Where;

$$\varepsilon =RT\ln(1+\frac{1}{C_{e}})$$

Where *Q*_*e*_ is the amount of metal ions adsorbed by biosorbent (mol/g), *C*_*e*_ is the concentration of metal ions in equilibrium (mol/L), *β* is the adsorption energy (kJ^2^/mol^2^) and *Q*_*m*_ is the maximum adsorption capacity (mol/g). The value of *β* and *Q*_*m*_ can be evaluated from slope and intercept of a plot of ln*Q*_*e*_ against *ε*^*2*^. The mean sorption energy (kJ/mol) can be written as:

$$E=\frac{1}{\sqrt{-2\beta }}$$ (19)

If the magnitude of *E* is in the range of 8 and 16 kJ/mol, the biosorption process is supposed to be chemisorption, if it is *E ≤* 8 kJ/mol then physisorption is the predominant mechanism.

### Flory-Huggins isotherm model

This isotherm explains the degree of surface coverage characteristics of adsorbate onto adsorbent as well as the feasibility and spontaneous nature of an adsorption process [45].

$$\log(\frac{C_{o}-C_{e})}{C_{o}}=\log\;K_{FH}-n_{FH}\;\log(\frac{C_{e}}{C_{o}})$$ (20)

Where, *K*_*FH*_ is the model equilibrium constant (L/mg) and *n*_*FH*_ its exponent. The standard Gibb's free energy (Δ*G°*) can be calculated as;

$$\Delta G^{o}=-RT\ln(K_{FH})$$ (21)

The value of standard Gibb's free energy may give the information about the endothermic or exothermic nature of biosorption process.

### Hill-der Boer isotherm model

The Hill-der Boer isotherm model expresses the interactions between adsorbate-adsorbate in the solid phase and adsorbate-adsorbent at the liquid-solid interface [46].

$$\frac{\theta }{1‐\theta }+\ln(\frac{\theta }{1‐\theta })-\ln C_{e}=-\ln k_{2}+k_{1}\theta$$ (22)

Where *θ*(1–*C*_*e*_/*C*_*o*_) is the surface coverage fraction, *k*_1_ (dimensionless) and *k*_2_ (mg/L) are constants respectively.

### Halsey isotherm model

Hasely isotherm model describes the multi-layer sorption of metal ions on heterogeneous biomass surface [47].

$$\ln\;Q_{e}=\frac{1}{n_{H}}\ln\;K_{H}-\frac{1}{n_{H}}\ln\;C_{e}$$ (23)

The values of *n*_*H*_ and *K*_*H*_ can be obtained from the slope and intercept of a linear plot between ln*Q*_*e*_ and ln*C*_*e*_.

### Fundamental thermodynamic parameters

Change in free energy of biosorption, which is a fundamental criterion of spontaneity of the process. The standard Gibbs free energy change (Δ*G*^*o*^) can be calculated from the Langmuir equilibrium constant at different temperatures [48].

$$\Delta G^{o}=-RT\;\ln(b)$$ (24)

The experimental data can be further analyzed to evaluate the change in standard enthalpy (Δ*H*^*o*^) and entropy (Δ*S*^*o*^) of biosorption process as a function of temperature using van’t Hoff equation.

$$\ln\;(b)=-\frac{\Delta H^{o}}{\mathrm{RT}}+\frac{\Delta S^{o}}{R}$$ (25)

The plot of ln*b* and 1/*T* gives a linear line, in which -Δ*H*^*o*/^/*R* and Δ*S*^*o*^/*R* are equal to its slope and intercept respectively.

## Results and discussion

### Evaluation of precision and accuracy of Cr(VI) concentrations

**Fig 2:**
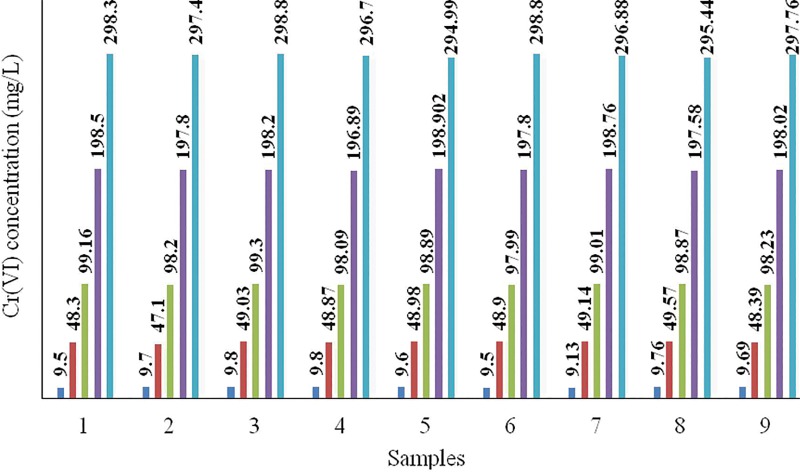
Quality control and number of samples.

The calibration curve was made in the linear range of Cr(VI) concentrations from 0.3 to 2 mg/L with absorbance between 0.159 and 1.137 using a spectrophotometer. The linear regression result of absorbance against Cr(VI) concentration was A = 0.5704C-0.0012 with correlation coefficient of 0.9988. Quality control (QC) samples against the number of samples taken are shown by Fig 2.

The precision is expressed by relative standard deviation and variance methods. Relative standard deviation (RSD) is a statistical measurement that describes the spread of data with respect to the mean, and the results are expressed in terms of percentage. The accuracy is determined as recovery percentage. The precision and accuracy results are presented in Table 1.

**Table 1:** Precision and accuracy data of Cr(VI) analyses.

QC samples (mg/L)	Average (mg/L)	SD	RSD (%)	Variance	Recovery (%)
10	9.60	0.219	2.219	0.045	96.088
50	48.697	0.709	0.456	0.502	97.395
100	98.637	0.579	0.512	0.255	98.637
200	198.05	0.627	0.350	0.393	99.025
300	297.238	1.369	0.460	1.876	99.076

### Effect of pH

**Fig 3:**
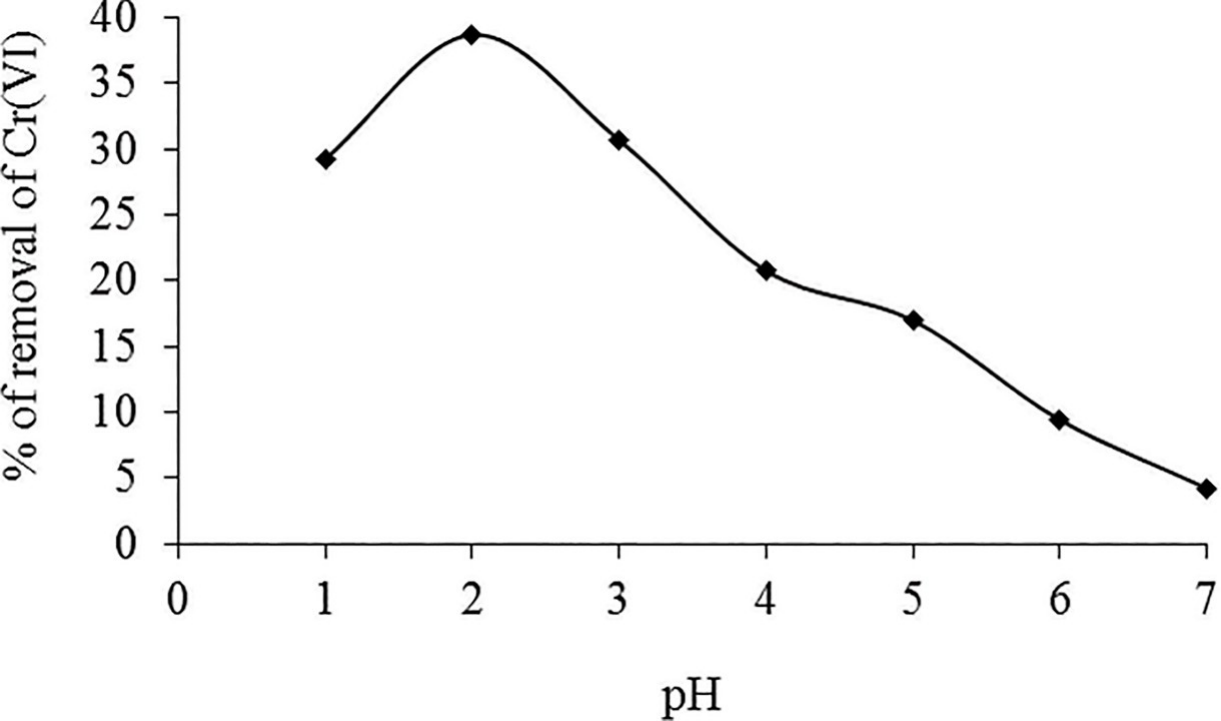
Effect of pH on biosorption of Cr(VI) by *Eupatorium adenophorum–*alginate beads at initial Cr(VI) concentration 10 mg/L and biomass concentration 1.0 g/L respectively.

The effect of pH on Cr(VI) biosorption onto *Eupatorium adenophorum*-alginate beads is shown in Fig 3. Indeed, the maximum removal of Cr(VI) ions was observed at pH 2.0. Under acidic conditions, the surface of the biomass becomes highly protonated and binds the Cr(VI) in the form of HCrO_4_^-^ ions [2,7]. Above pH 2.0, the gradual increase in negatively charged biomass surface groups as well as shifting of monovalent HCrO_4_^-^ to divalent Cr_2_O_7_^-2^ and CrO_4_^-2^ ions in aqueous solutions resulted the decrease in Cr(VI) biosorption efficiency [1,6,7,49]. The similar trends were also observed for Cr(VI) biosorption in various plant biosorbents [3,5,6,10,23,50].

### Effect of biomass concentration

**Fig 4:**
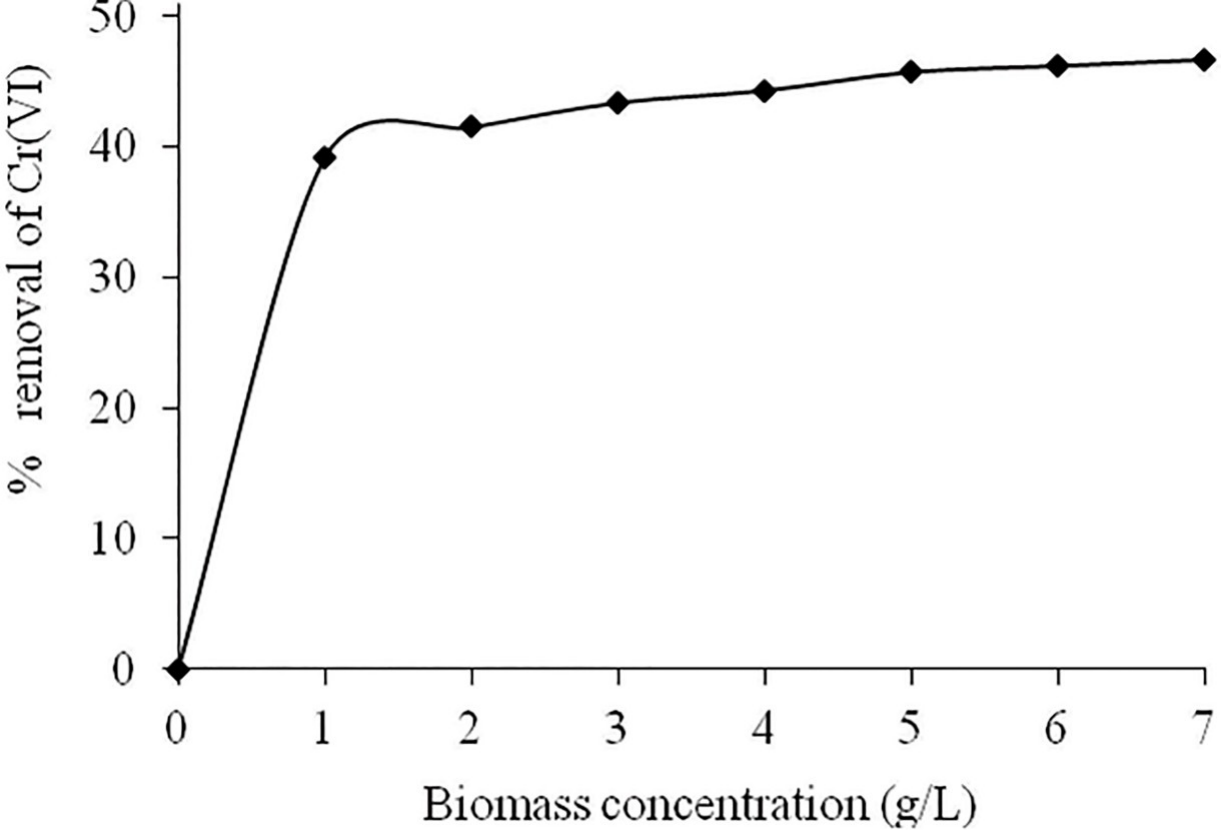
Effect of biomass concentration on biosorption of Cr(VI) by *Eupatorium adenophorum*–alginate beads at initial Cr(VI) concentration 10 mg/L and pH 2.0 respectively.

As shown in Fig 4, the removal efficiency of Cr(VI) ions was increased with increasing the biomass concentrations from 1.0 to 7.0 g/L respectively. This could be ascribed to the availability of more binding sites of *Eupatorium adenophorum* biomass for Cr(VI) ions [10,23]. On the contrary, the uptake capacities of Cr(VI) ions found to be decreased from 3.915 to 0.667 mg/g with increase in biomass concentrations from 1.0 to 7.0 g/L (Fig is not shown). It may be due to the fact that *Eupatorium adenophorum*-alginate beads has a limited number of active binding sites for Cr(VI) ions, and it could be achieved the saturation above a certain biomass concentration [6,51].

### Effect of contact time and temperature

**Fig 5:**
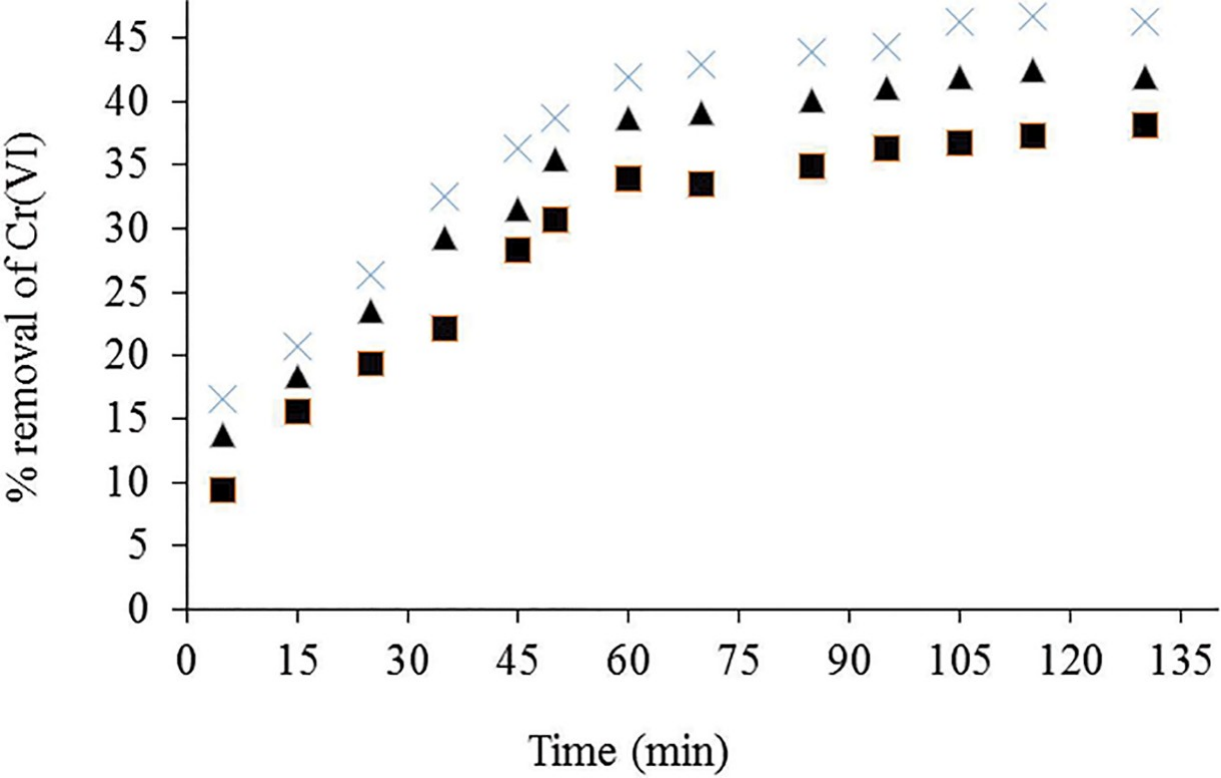
Effect of contact time on Cr(VI) biosorption by *Eupatorium adenophorum*–alginate beads at initial Cr(VI) concentration 10 mg/L, pH 2.0 and biomass concentration 1.0 g/L at 20 (-■—), 30 (-×-) and 40°C (-▲-) respectively.

The effect of contact time for Cr(VI) biosorption on *Eupatorium adenophorum*-alginate beads at different temperatures is shown in Fig 5. The graphs show that the biosorption efficiency of Cr(VI) ions increases with increase in contact time. A contact time of 60 min was sufficient to achieve equilibrium. The results revealed that the removal efficiency of Cr(VI) ions was not significantly changed with further progress of contact time. On the other hand, Cr(VI) bisorption efficiency was increased with increase in temperature from 20 to 30 ^o^C. This may be due to the increase in collision frequency between Cr(VI) ions and the biomass species [10]. It was also found that the removal efficiency of Cr(VI) ions was slightly decreased above 30°C, suggesting the destruction of some binding sites [22,23].

### Effect of initial Cr(VI) concentrations

**Fig 6:**
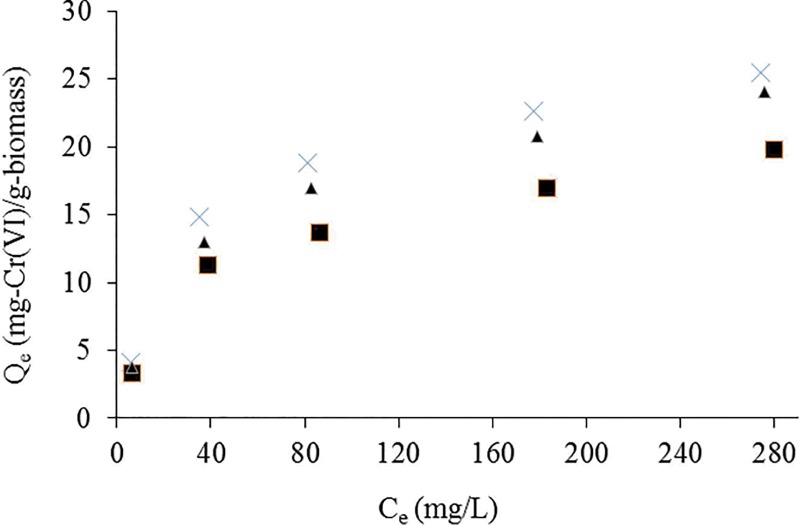
Effect of initial concentration of Cr(VI) ions onto *Eupatorium adenophorum*-alginate beads at initial Cr(VI) concentration from 10 to 300 mg/L, pH 2.0, contact time 60 min and biomass concentration 1.0 g/L at 20 (-■—), 30 (-×-) and 40°C (-▲-) respectively.

Effect of initial Cr(VI) concentrations on equilibrium uptake capacity at different temperatures is shown in Fig 6. It was found that the amount of equilibrium biosorption capacity increases with increase in initial Cr(VI) concentrations. High uptake capacity of Cr(VI) ions was observed at lower Cr(VI) concentrations. This may be due to the sufficient binding sites available on biomass surface. At higher concentrations, relatively less available binding sites may responsible for reduction of uptake capacity of Cr(VI) species by *Eupatorium adenophoru*-alginate beads [52]. Similar results have been reported by many authors using different types of plant biosorbents [3,10,39,52].

### Determination of kinetic parameters

Biosorption kinetics can play a vital role to select and design the reactor systems [2]. Various kinetic models have been used to determine the biosorption rate of heavy metals on biomass surface. The experimental data were determined to be well predicted by models with correlation coefficient values closer to unity [5]. Kinetic constants and correlation coefficients of kinetic models are given in Table 2.

**Table 2:** Kinetic parameters of Cr(VI) sorption on *Eupatorium adenophorum*–alginate beads at initial Cr(VI) concentration 10 mg/L, pH 2.0 and biomass concentration 1.0 g/L respectively.

**Kinetic model**	**Parameter**	**Temperature**		
		**20°C**	**30°C**	**40°C**
	*Q*_e,exp_ (mg/g)	3.820	4.669	4.198
**Langmuir-Hinshelwood**	*k*_*1*_ (min^-1^)	0.008	0.001	0.009
	*k*_*0*_ (L/mg)	0.103	0.108	0.107
	*k*_1_/*k*_*0*_ (mg/L.min)	0.007	0.010	0.0083
	*R*^*2*^	0.848	0.857	0.835
**Avrami**	*n*	0.861	0.743	0.811
	*k*_*av*_ (min^-1^)	0.082	0.180	0.126
	*R*^*2*^	0.961	0.936	0.933
**Lagergren pseudo-first order**	*k*_*1*_ (min^-1^)	0.057	0.052	0.0529
	*Q*_*e*,_ (mg/g)	6.382	5.353	5.228
	*R*^*2*^	0.877	0.931	0.921
**Ritchie pseudo second-order**	*Q*_*e*_(mg/g)	4.486	3.861	4.163
	*k*_*2*_ (min^-1^)	0.105	0.060	0.088
	*R*^*2*^	0.859	0.950	0.892
**Sobkowsk and Czerwinski pseudo** **second-order**	*k*_*2*_ (min^-1^)	0.302	0.260	0.397
	*R*^*2*^	0.790	0.900	0.753
**Blanachard pseudo second-order**	*k*_*2*_ (g/mg min)	0.079	0.0563	0.0946
	α (g/mg)	1.758	0.809	1.884
	*R*^*2*^	0.830	0.890	0.901
**Ho pseudo second-order**	*k*_*2*_ (gm/g min)	0.0079	0.0085	0.0084
	*h* (mg/g)/min	0.161	0.259	0.220
	*Q*_*e*_ (mg/g)	4.710	5.494	5.107
	*R*^*2*^	0.980	0.988	0.987
**Elovich**	α (mg/g.min)	2.700	5.143	3.214
	*β* (g/mg)	1.054	2.313	1.537
	*R*^*2*^	0.952	0.953	0.953
**Intraparticle diffusion**	*k*_*id*_ (mg/g min^0.5^)	0.327	0.354	0.336
	*C* (mg/g)	0.422	1.041	0.819
	*R*^*2*^	0.938	0.932	0.928

The best correlated kinetic data with Langmuir-Hinshelwood (L-H) kinetic model gives high correlation coefficient values, which indicates the chemisorption as a predominant mechanism [29]. The low *R*^2^ values at different temperatures showed that this model can not be applied to predict the biosorption kinetics. However, Cr(VI) biosorption may be proceeded with physisorption mechanism [12].

Avrami model describes the possible changes of biosorption mechanism with multiple kinetic orders [30]. In this study, low *R*^2^ values indicated that kinetic data of Cr(VI) biosorption on *Eupatorium adenophorum-*alginate beads were not agreement with Avrami model.

The fitness of Cr(VI) biosorption data with low correlation coefficients as well as lower *Q*_e,cal_ comparison to *Q*_e,exp_ indicated that Cr(VI) biosorption on *Eupatorium adenophorum-*alginate beads did not follow Lagergren pseudo-first order reaction [12].

The results showed that the regression (*R*^*2*^) values of Ho pseudo second order model were nearly unity. Results confirmed that biosorption data were well represented by Ho pseudo second order model. Additionally, it was also found that the *Q*_e,exp_ values were good agreement with the *Q*_e,cal_ ones. The rate constant (*k*_2_) and initial sorption rate (*h*) values were higher at 30 ^o^C comparison to the other studied temperatures, indicating that the biosorption of Cr(VI) species becomes faster at 30 ^o^C [32].

The comparatively low correlation coefficient values were observed with Ritchie, Sobkowsk and Czerwinski, and Blanachard pseudo second-order kinetic models respectively. Results indicated that biosorption process was not well described with these kinetic models [12].

The best correlation of experimental kinetic data to the Elovich kinetic model is the evidence of chemisorption process. Relatively low *R*^*2*^ values at all temperature was not suggested this model for Cr(VI) biosorption onto *Eupatorium adenophorum*-alginate beads [36].

**Fig 7:**
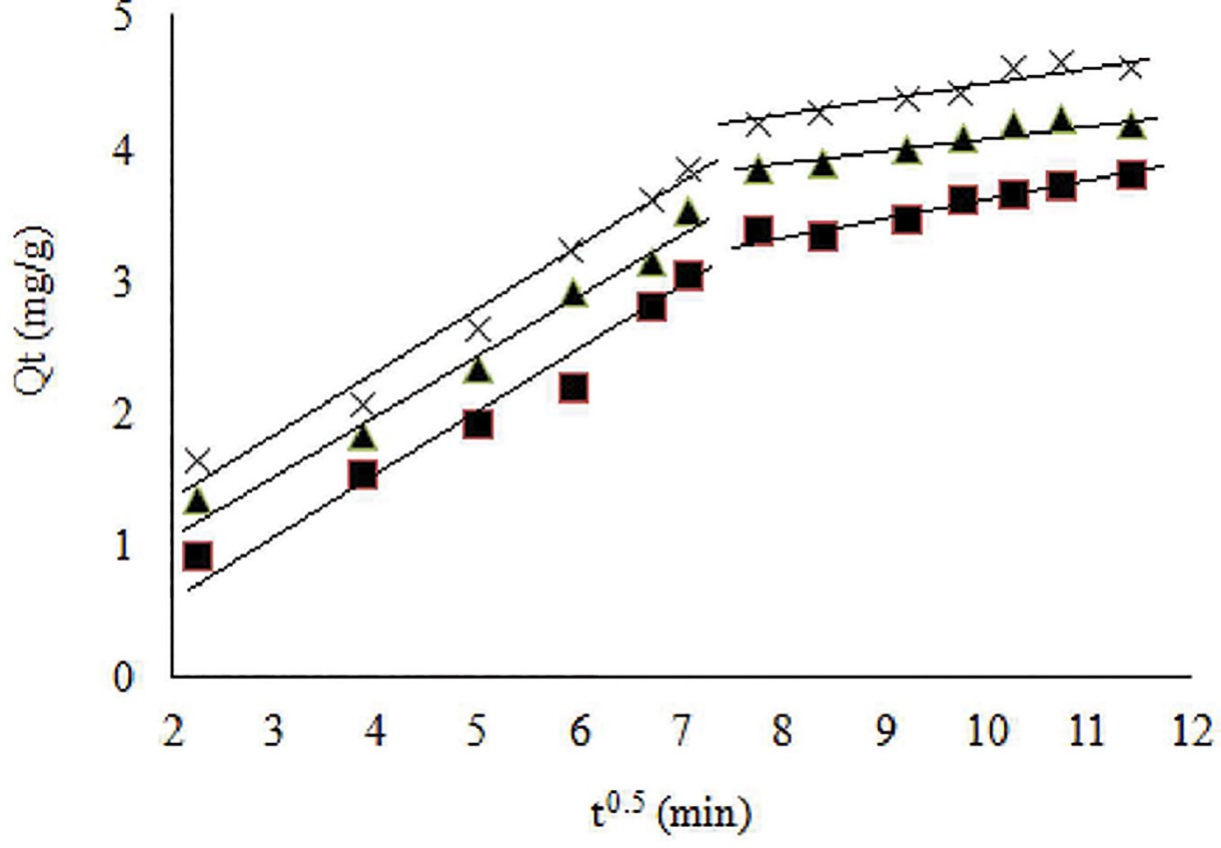
Intraparticle diffusion plots of Cr(VI) biosorption at initial concentration 10 mg/L, pH 2.0 and biomass concentration 1.0 g/L at 20 (-■—), 30 (-×-) and 40°C (-▲-) respectively.

Intraparticle diffusion model identifies the diffusion mechanisms and rate controlling steps affecting the biosorption process. The smaller values of *R*^*2*^ evidenced that experimental kinetic date were not fitted the model very well. It was also found that values of *k*_*id*_ increased with increasing temperature from 20 to 30 ^o^C and then decrease to 40 ^o^C. Larger values of *k*_*id*_ may suggest the faster diffusion. On the contrary, increase in values of *C* indicated the increase in the thickness of the boundary layer and decrease in chance of the external mass transfer. The plots of *Q*_*t*_ versus *t*^*0*.*5*^ at different temperatures are given in Fig 7. The multi-linearity of plots indicated that Cr(VI) biosorption may take place in multiple steps [5,6]. First linear portions may suggest the boundary layer diffusion of Cr(VI) ions, whereas those of second portions may suggest the intraparticle diffusion starts to slow down due to the extremely low Cr(VI) concentration left in the biosorption medium [37].

### Biosorption isotherms

The extent of biosorption can be correlated in terms of an adsorption isotherm and it explains the interaction between the adsorbate and adsorbent in biosorption process. The isotherm models parameters along with regression coefficients on Cr(VI) biosorption were investigated and the results are listed in Table 3.

**Table 3:** Isotherm parameters of Cr(VI) sorption on *Eupatorium adenophorum*–alginate beads at initial Cr(VI) concentration from 10 to 300 mg/L, pH 2.0 and biomass concentration 1.0 g/L respectively.

Isotherm model	Parameter	Temperature		
		20 ^o^C	30 ^o^C	40 ^o^C
**Langmuir**	*Q*_*max*_ (mg/g)	20.611	28.011	25.575
	*b* (L/mg)	0.0291	0.0292	0.0272
	*K*_*L*_	0.109–0.837	0.111–0.853	0.117–0.854
	*R*^*2*^	0.997	0.999	0.999
**Scattered**	*Q*_*max*_ (mg/g)	21.035	27.744	26.007
	*b* (L/mg)	-0.027	-0.029	-0.026
	*R*^*2*^	0.964	0.984	0.982
**Freundlich**	*K*_*f*_ (L/g)	1.609	2.212	1.773
	*n*	2.151	2.119	2.055
	*R*^*2*^	0.946	0.938	0.951
**Gin**	*α*	0.899	0.862	0.876
	*β* (mg/L.g)	-3.168	-4.014	-3.520
	*R*^*2*^	0.855	0.818	0.843
**Temkin**	*b*_*T*_ (kJ/mol)	0.574	0.460	0.495
	*a*_*T*_ (L/mol)	0.332	0.382	0.317
	*R*^*2*^	0.993	0.997	0.997
**D-R**	*Q*_*m*_ (mg/g)	51.879	70.275	66.879
	*E* (kJ/mol)	9.128	10	10
	*R*^*2*^	0.978	0.965	0.978
**Hill-der Boer**	*k*_*1*_	21.981	19.089	20.559
	ln*k*_*2*_	9.391	9.391	9.468
	*R*^*2*^	0.993	0.994	0.996
**Flory-Huggins**	*n*_*FH*_	-0.317	-0.283	-0.306
	*K*_*FH*_ (L/mg)	0.992	0.989	0.990
	Δ*G*^*o*^ (kJ/mol)	-18.513	-25.695	-26.022
	*R*^*2*^	0.955	0.955	0.942
**Halsey**	*n*_*H*_	-2.151	-2.133	-2.055
	*K*_*H*_ (L/mg)	0.359	0.198	0.307
	*R*^*2*^	0.946	0.932	0.951

**Fig 8:**
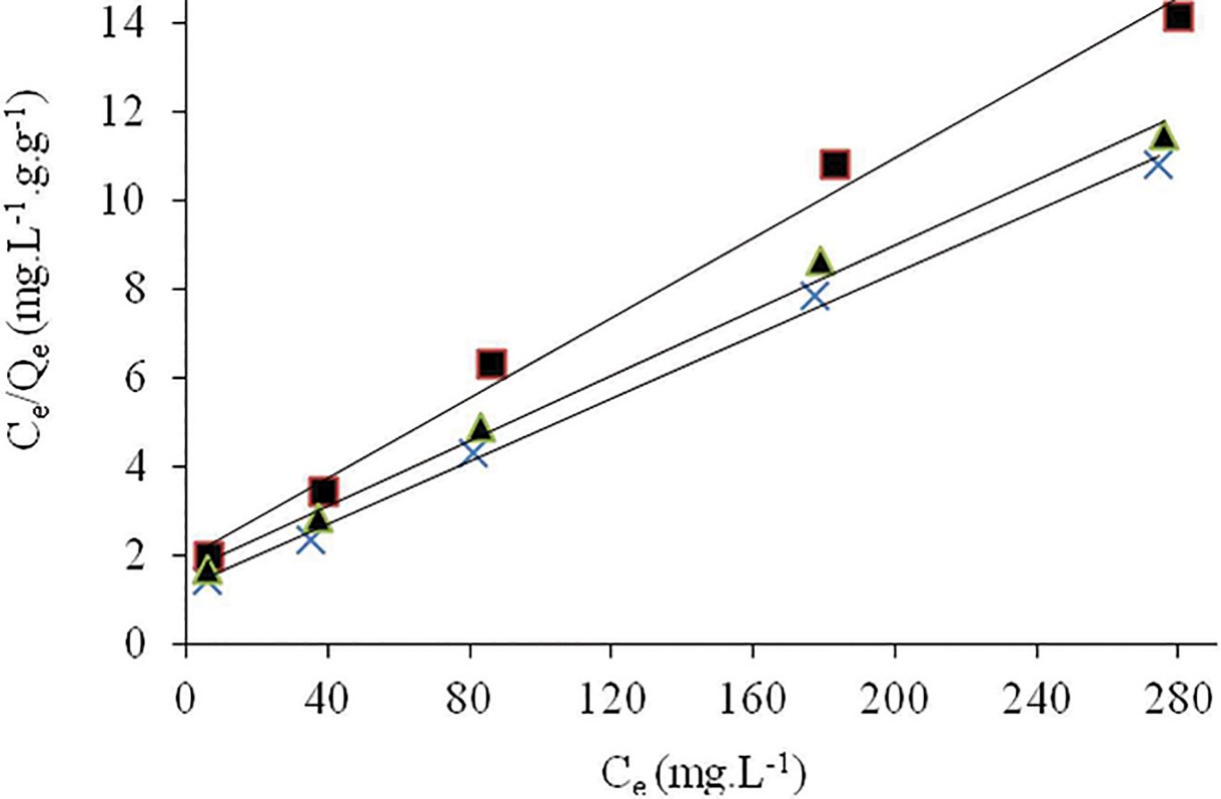
Langmuir isotherm for Cr(VI) ions onto *Eupatorium adenophorum*–alginate beads at initial Cr(VI) concentration from 10 to 300 mg/L, pH 2.0, contact time 60 min and biomass concentration 1.0 g/L at 20 (-■—), 30 (-×-) and 40°C (-▲-) respectively.

The application of Langmuir isotherm plots for Cr(VI) biosorption at different temperatures is shown in Fig 8. The maximum uptake capacity of *Eupatorium adenophorum*-alginate beads for Cr(VI) was calculated at 28.011 mg/g at optimum conditions. The comparative analysis of *R*^*2*^ values indicated that the Cr(VI) biosorption was best described by Langmuir model. It may be attributed to the homogeneous distribution of binding sites on biomass surface and mono-layer sorption of Cr(VI) species [6]. It also suggested that there was no interaction between Cr(VI) ions adsorbed on neighboring binding sites. Moreover, *K*_*L*_ values were between 0 and 1, which confirmed the viability of Cr(VI) biosorption on *Eupatorium adenophorum-*alginate beads [39]. A comparison of maximum uptake capacity of Cr(VI) on *Eupatorium adenophorum-*alginate beads with different plant biosorbents reported in the literature is presented in Table 4.

**Table 4:** Comparison of Cr(VI) uptake capacity with other plant biosorbents.

Biosorbent	*Q*_*max*_ (mg/g)	Reference
***Cupressus lusitanica* bark**	305.4	[10]
***Senna siamea* seed**	139.86	[6]
***Cicer arientinum***	72.16	[14]
**Mango peels**	66.66	[2]
***Polyporus squamosus***	31.2	[19]
***Eupatorium adenophorum*-alginate beads**	28.011	Present study
***Stipa tenacissima* L**	18.51	[3]
***Eichhorniacrassipes***	6.0	[53]
***Plataneusorientalis* leaves**	5.01	[18]
***Euclea schimperi***	3.946	[13]
***Acacia albida***	2.983	[13]
**Wheat bran**	0.942	[21]
***Ulmus* leaves**	0.9	[20]

**Fig 9:**
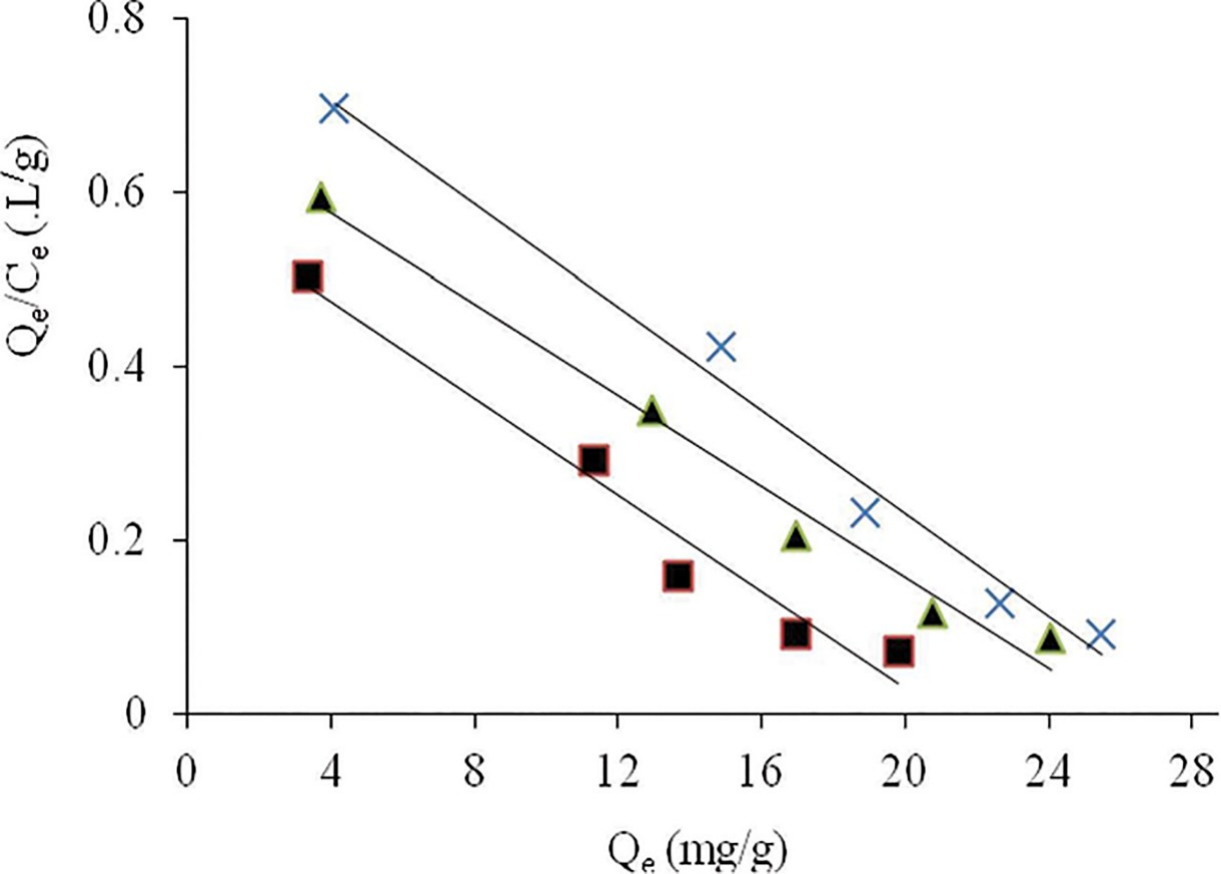
Scatchard plots for Cr(VI) ions onto *Eupatorium adenophorum–*alginate beads at initial Cr(VI) concentration from 10 to 300 mg/L, pH 2.0, contact time 60 min and biomass concentration 1.0 g/L at 20 (-■—), 30 (-×-) and 40°C (-▲-) respectively.

Fig 9 shows the Scatchard plots for Cr(VI) biosorption on *Eupatorium adenophorum-*alginate beads at different temperatures. The higher values of *R*^*2*^ indicated the satisfactory fitting of the experimental data. It confirmed that the binding sites of this biosorbent exhibited the same affinity towards Cr(VI) ions, and further supported the mono-layer biosorption process as described by Langmuir isotherm model [40].

Freundlich isotherm model gave a less reasonable fit to the experimental data comparison to Langmuir model, indicating that biosorption of Cr(VI) ions was not followed by this isotherm model. However, values of Freundlich constant, *n* in the range of 2 to10 suggested that Cr(VI) sorption was favorable on *Eupatorium adenophorum*-alginate beads [41].

The fairly low linear regression (*R*^*2*^) values were not ascertained the good fitness of equilibrium data for Gin isotherm model. But, the negative values of *β* confirmed the feasibility of Cr(VI) biosorption on *Eupatorium adenophorum*-alginate beads [42].

The high values of *R*^*2*^ obtained from equilibrium data showed the applicability of Temkin isotherm model for Cr(VI) biosorption on *Eupatorium adenophorum*-alginate beads. The values of heat of biosorption, *b*_*T*_ are positive at all temperatures, which may suggest the Cr(VI) biosorption was endothermic in nature. On the other hand, lower values of *b*_*T*_ suggested the weak interaction between Cr(VI) ions and binding sites [43].

In all cases, Dubinin-Radushkevich (D-R) isotherm model exhibited lower correlation coefficient values. Also, the values of maximum uptake capacities determined were much higher than those of experimental values. Therefore, D-R isotherm model was unable to describe the Cr(VI) biosorption onto *Eupatorium adenophorum*-alginate beads. In addition, the numerical values of *E* suggested the physisorption was predominant mechanism for Cr(VI) biosorption [6,44].

The degree of surface coverage of the biomass can be studied by Flory-Huggins model. The correlation coefficient values may imply the moderate applicability of Cr(VI) biosorption. Negative values of Gibbs free energy indicated the spontaneous nature and feasibility of Cr(VI) biosorption on *Eupatorium adenophorum*-alginate beads [12,45].

High correlation coefficient values revealed that Hill-der Boer model is the best prediction of Cr(VI) biosorption on *Eupatorium adenophorum*-alginate beads. The strong interaction between Cr(VI) ions and *Eupatorium adenophorum* surface binding sites was supported by the higher values of *k*_1_ and lower values of *k*_2_ respectively [46].

Hasely isotherm model describes the surface heterogeneity of biomass surface and multi-layer biosorption process. The calculated values of correlation coefficient indicated that multi-layer sorption was not involved in Cr(VI) biosorption on *Eupatorium adenophorum*-alginate beads [47].

### Thermodynamic analysis

The fundamental thermodynamic parameters for biosorption of Cr(VI) ions on *Eupatorium adenophorum*-alginate beads at different temperature are depicted in Table 5. The negative values of Δ*G*^*o*^ obtained at all temperatures confirmed the spontaneous and feasibility nature of Cr(VI) biosorption process [3,5,6]. Magnitudes of Δ*G*^*o*^ suggested the Cr(VI) biosorption on *Eupatorium adenophorum*–alginate beads imply the physisorption mechanism [54]. Positive value of Δ*H*^*o*^ showed the endothermic nature of biosorption process [2,3]. In addition, the calculated values of Δ*H*^*o*^ further supported to the physisorption as operation phenomenon [55]. Moreover, the positive value of entropy change (Δ*S*^*o*^) indicated an increase in the randomness at the solid/solution interface during the biosorption process [3].

**Table 5:** Thermodynamic parameters of Cr(VI) biosorption onto *Eupatorium adenophorum*-alginate beads.

*C*_*o*_ (mg/L)	*T* (^o^C)	Thermodynamic parameters		
		Δ*G*^*o*^ (kJ mol^−1^)	Δ*H*^*o*^ (kJ mol^−1^)	Δ*S*^*o*^ (J mol^−1^K)
10	20–40	-18.156 to-18.323	2.612	53.549

### Effect of interfering co-ions

The co-ions present in wastewater may compete with primary metal ions on limited binding sites, which can reduce the biosorption performance in operating systems [29]. Table 6 shows the effect of co-existing ions on Cr(VI) biosorption onto *Eupatorium adenophorum*-alginate beads. Results demonstrated that Cr(VI) biosorption performance was not significantly affected by the presence of Mg^+2^, Ca^+2^, Zn^+2^, Cd^+2^, Cu^+2^ and Ni^+2^ ions in studied concentrations, suggesting that such ions were unable to chelate the surface binding sites [12]. On the other hand, Cr(VI) biosorption efficiency was found to be slightly decreased with increase in concentration of SO_4_^—^, Cl^-^, and CO_3_^-^ ions, indicating the competitive phenomenon of same charged species in the same binding sites [29]. Furthermore, sorption efficiency of Cr(VI) increased with increasing the concentration of Fe^+3^ ions. This may be attributed to the Fe^+3^ ions on biomass surface may generate the new binding sites for Cr(VI) ions on biomass surface [56].

**Table 6:** Effect of interfering ions on Cr(VI) biosorption at initial concentration of 10 mg/L at optimum conditions.

Co-ions (mg/L)	Cr(VI) removal (%)									
	SO_4_^-2^	Cl^-^	CO_3_^-2^	Mg^+2^	Ca^+2^	Fe^+3^	Zn^+2^	Cd^+2^	Cu^+2^	Ni^+2^
5	41.10	43.53	43.08	38.18	38.22	43.08	41.08	44.55	41.09	36.01
10	40.03	38.18	40.58	38.03	39.81	46.77	41.75	38.99	38.50	38.55
25	41.61	38.97	41.12	40.89	41.38	51.04	39.65	42.05	40.28	39.11
50	37.78	36.71	36.97	42.66	38.74	59.33	40.47	42.13	42.00	42.67

### Desorption studies

The desorption of Cr(VI) ions from metal-loaded *Eupatorium adenophorum*-alginate beads was observed at 92.091% using 0.5 M HNO_3_ solution at room temperature. High desorption efficiency of Cr(VI) ions from the biomass surface within a short period may confirm the weak interaction between Cr(VI) ions and binding sites [56]. Relatively low desorption efficiency (65%) of Cr(VI) ions from *Casurina equisetifolia* biomass has also reported by Ranganathan, [40].

## Conclusions

Calcium alginate entrapped *Eupatorium adenophorum* Spreng.stems powder biomass showed the high removal efficiency for removal of Cr(VI) species from aqueous solutions. It was found that biosorption kinetics fitted well with pseudo-second order among different kinetic models owing to high *R*^*2*^ values. Due to high values of the correlation coefficient, sorption equilibrium data followed by Langmuir, Temkin and Hill-der Boer isotherm as compared to other isotherm models. Scatchard plots analysis indentified the mono-layer biosorption process as described by Langmuir isotherm model. Cr(VI) biosorption was not significantly affected by the presence of interfering co-ions at lower concentrations. Thermodynamic parameters values demonstrated that the endothermic, spontaneous and feasibility nature of Cr(VI) biosorption on *Eupatorium adenophorum*-alginate beads. Temkin and Dubinin-Radushkevich isotherm models as well as Δ*G*^*o*^ and Δ*H*^*o*^ values at all the studied temperatures suggested that physisorption was the predominant mechanism for Cr(VI) biosorption. High desorption efficiency of Cr(VI) ions from the Cr(VI)-loaded biomass using dilute HNO_3_ solution within a short period may confirm the weak interaction between Cr(VI) ions and biomass binding sites It can be concluded from these results that *Eupatorium adenophorum*-alginate beads can be used as an efficient and eco-friendly biosorbent for the Cr(VI) removal from aqueous media.
